# Development of a novel carrier optimized for cell sheet transplantation

**DOI:** 10.1080/21592535.2015.1027846

**Published:** 2015-04-14

**Authors:** Yosuke Amagai, Kaoru Karasawa, Jung Kyungsook, Akira Matsuda, Masanori Kojima, Jun Watanabe, Toyoji Hibi, Hiroshi Matsuda, Akane Tanaka

**Affiliations:** 1 Cooperative Major in Advanced Health Science; Graduate School of Bio-Applications and System Engineering; Laboratories of; 2 Comparative Animal Medicine and; 3 Veterinary Molecular Pathology and Therapeutics; Division of Animal Life Science; Tokyo University of Agriculture and Technology; Tokyo, Japan; 4 Nikkan Industries Co.; Ltd.; Tokyo, Japan; 5 Sakado plant; Tokyo, Japan

**Keywords:** Carrier, cell sheet, cornea, poly(lactic acid), transplantation

## Abstract

Tissue engineering is a rapidly advancing technology in the field of regenerative medicine. For the transplantation of cell sheets, a carrier must maintain the shape of a cell sheet from a culture dish to affected sites as well as release the sheet easily onto the lesion. In this study, we examined the utility of a novel, poly(lactic acid)-based carrier for cell sheets transplantation to the cornea of dogs and the skin of rats. The poly(lactic acid)-based carrier easily picked a cell sheet up from the dish, fit to the shape of the transplantation sites, and saved time for cell sheets detachment comparing to a conventional carrier. Thus, the poly(lactic acid)-based carrier would be useful for easy cell sheet transplantations.

## Abbreviations

α-MEM: α-minimum essential medium
FBS: fetal bovine serum
PBS: phosphate-buffered saline

## 

Tissue engineering approaches are widely used to develop functional substitutes for damaged tissues and organs through cellular transplantation.^1^ Applications for cell sheet technology have been actively investigated in this field, as represented by preclinical or clinical studies of cell sheets derived from corneal cells, myocardial cells, and keratinocytes.^2-6^ For the transplantation of cell sheets from a culture dish to the affected site, a carrier that can maintain its morphology without any shrinkage is necessary. Recently, membranes consisting of poly(vinylidene difluoride) or nitrocellulose have been developed,^2^ although there are 2 main difficulties associated with these methods. First, because the membranes are rigid, they do not easily fit the curved sites properly. Second, detaching cell sheets from the membrane requires careful treatment and is a time-consuming process. Other materials have also been used recently;^7,8^ however, improvements in the flexibility and rapidity of cell sheet detachment are still required.

To resolve these problems, we developed a novel, poly(lactic acid)-based carrier. The use of the poly(lactic acid) can minimize the safety hazards because the material has been used in biomedical areas for several applications for decades.^9^ In this study, we examined the characteristics of the carrier and applied it to the surfaces of a dog cornea as well as rat dermis to evaluate its practical utility. All experiments using animals complied with the standards specified in the guidelines of the University Animal Care and Use Committee of the Tokyo University of Agriculture and Technology.

**Figure 1.**
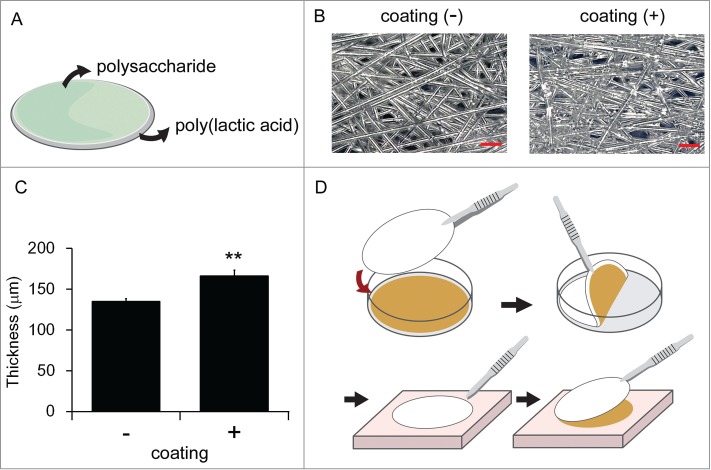
Schema of the poly(lactic acid)-based carrier (**A**). Photographs of the surface of carrier in the presence or absence of the coating (**B**). The images were taken by using VHX-5000 digital microscope (Keyence Corp.). Original magnification, ×400. Bar; 100 μm. Comparison of coating-free and coated carrier thicknesses (**C**). The experiment was conducted 3 times and the mean ± SE is indicated. ** *P*< 0.01 by one-way ANOVA. A procedure of the carrier application (**D**). The carrier adhered to cell sheets after being overlaid on the sheets for approximately 5 min. After transfer of cell sheets to the affected sites, dropping of pre-warmed physiological solution onto the carrier allowed cell sheets detachment.

The novel carrier is made with 30 g/m^3^ poly(lactic acid) sheet coated with a mixture of 60% pectin, 20% glycerin, 15% starch, and others (e.g. agarose and emulsifying agents) (**Fig. 1A and 1B**). Thickness of the coating is approximately 30 μm, which was measured by comparing the thickness of coating-free and coated poly(lactic acid) sheets (**Fig. 1C**). It binds firmly to cell sheets at room temperature, enabling easy transfer of the cell sheet to the affected site. Because the coating is soluble in water at approximately 40°C, the cell sheets could easily detach from the carrier by dropping a warm liquid such as saline onto the carrier (**Fig. 1D**).

**Figure 2.**
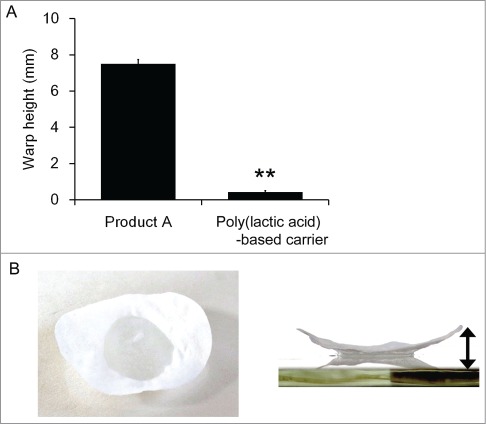
Comparison of the warp height between poly(lactic acid)-based carrier and Product A (**A**). The experiment was conducted 3 times and the mean ± SE is indicated. **P < 0.01 compared to Product A by one-way ANOVA. Representative data of warping in Product A (**B**). The arrow indicates the warp height.

Some materials automatically alter their shape as they get wet, which is not an ideal property for a cell sheet carrier in terms of its flexibility. Thus, the warp height resulting from PBS absorption was measured using the poly(lactic acid)-based carrier and a commercial carrier (Product A). Each carrier was placed on a drop of PBS on the glass for 10 sec, and the warp height was measured using a height gage at room temperature. In consequence, the poly(lactic acid)-based carrier was barely curved by the absorption of PBS, while Product A became warped with a height of approximately 8 mm (**Fig. 2A and 2B**). It indicates that the poly(lactic acid)-based carrier could easily fit the shape of transplantation sites.

**Figure 3.**
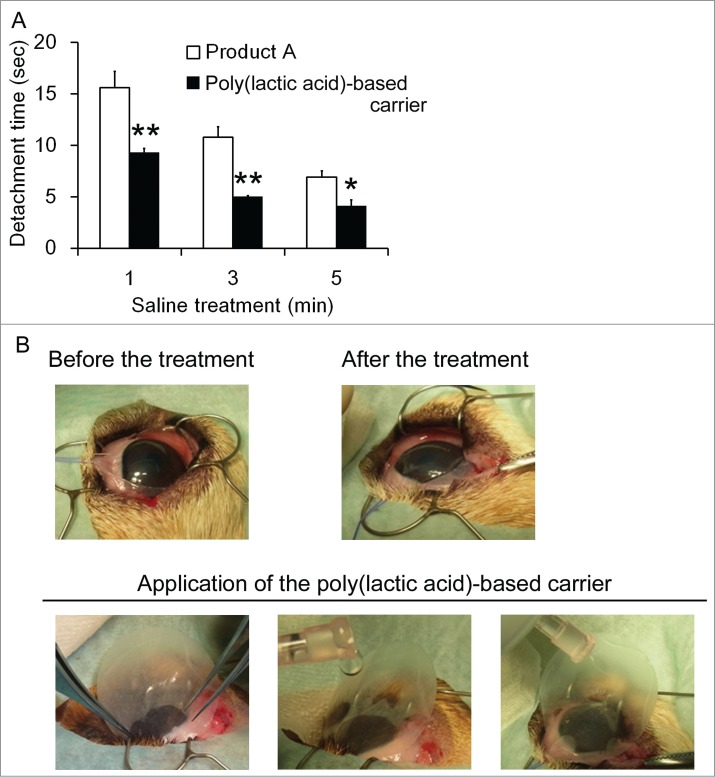
Comparison of the required time for cell sheets detachment in experimental transplantation of cell sheets onto the cornea (**A**). Each carrier along with cell sheets was applied onto the cornea, and pre-warmed saline (isotonic sodium chloride solution) was dropped from the upper part of each carrier for the indicated times and the duration required for cell sheet detachment was compared. The experiment was conducted 3 times and the mean ± SE is indicated. *P < 0.05, **P < 0.01, compared to Product A by one-way ANOVA. Representative image of cell sheet transplantation onto the surface of the canine cornea (**B**).

Next, cell sheets derived from dog adipose tissue were applied to the surface of the dog cornea. For the generation of cell sheets, approximately 1 g of adipose tissues was obtained from the dorsal subcutaneous fat of dogs under anesthesia with isoflurane. The tissue was digested using 0.1% collagenase (Wako) for 1 h to form single-cell suspensions, and then washed and cultured in α-MEM (Life Technologies) supplemented with 10% FBS (Hyclone) and antibiotics. After expansion, cells were trypsinized and cultured on an Upcell dish (CellSeed) until becoming confluent, and the resulting cell sheets were utilized for the transplantation. In this experiment, cell sheets that adhered to either the poly(lactic acid)-based carrier or Product A were applied and 40–42°C saline was dropped over the carrier for 1–5 min, and then the time required for cell detachment was compared. In this experiment, isotonic sodium chloride solution was used to detach cell sheets from the carrier. During the treatment, the dog was treated with retrobulbar anesthesia with 2% lidocaine and 0.5% bupivacaine under systemic anesthesia with isoflurane. The cell sheets detached from the poly(lactic acid)-based carrier faster than from Product A, regardless of the duration of pre-warmed saline treatment (**Fig. 3A**). Moreover, application of the sheet on the surface of the cornea was much easier when using the poly(lactic acid)-based carrier due to its pliability (**Fig. 3B**).

**Figure 4.**
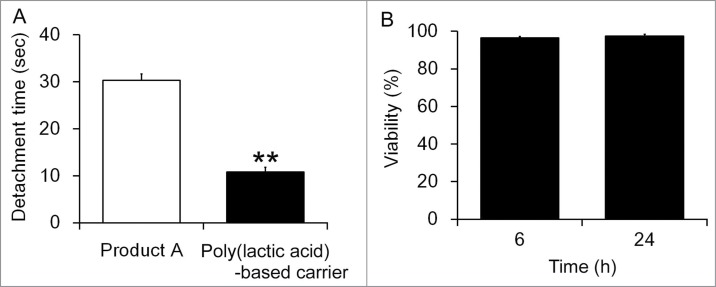
Comparison of the required time for cell detachment in experimental transplantation of cell sheets onto the rat dermis (**A**). Each carrier along with cell sheets was applied onto the dermis, and pre-warmed saline (isotonic sodium chloride solution) was dropped from the upper part of each carrier for 5 min and the duration required for cell sheet detachment was compared. The experiment was conducted 3 times and the mean ± SE is indicated. **P < 0.01 compared to Product A by one-way ANOVA. Cell viabilities of rat cells which was detached from the carrier (**B**). Rat cell sheets were bound to the carrier, treated with saline (isotonic sodium chloride solution) at 42°C for 5 min, cultured in the medium for indicated time and cell viabilities were determined. The experiment was conducted 3 times and the mean ± SE is indicated.

To validate whether these results would be applicable to other transplantation of cell sheets as well, cell sheets derived from rat adipose tissues were applied to the rat dermis. For the production of cell sheets, adipose tissues were obtained from the dorsal subcutaneous fat of 10-week-old male Wistar rats (Jcl:Wistar; Clea Co., Ltd.), and cultured according to the method used for dog adipose tissue as described above. The dorsal area of 10-week-old male Wistar rats (Jcl:Wistar) was shaved and the skin was removed with 8 mm disposable biopsy punches (Kai industries co., Ltd.) under anesthesia by isoflurane, and then the cell sheets were applied to the dermis. As a result, the poly(lactic acid)-based carrier detached from cell sheets faster than Product A corresponding to the results observed for the dog adipose-derived cells (**Fig. 4A**). The increased pliability of the carrier was also confirmed in this experiment. These results suggest that the carrier is a preferable tool for cell sheet transplantation.

Finally, we evaluated the toxicity of the coating in cell sheets. The rat adipose tissue sheets were bound to the carrier, treated with saline (isotonic sodium chloride solution) at 42°C for 5 min, cultured in α-MEM containing 10% FBS and antibiotics for 6 and 24 h and determined the cell viability. As shown in Fig. 4B, the viability of the cells was >95%, indicating the treatment with warm saline as well as remaining of the coating cause little cell damages.

Though further preclinical examinations are required to confirm its clinical utility, the risk of adverse effects by using our novel carrier is expected to be low because poly(lactic acid) is widely used in the medical field and the polysaccharides contained in the coating are also harmless to the human body. Because cell sheet therapy has a wide range of applications and shows potential to overcome currently incurable diseases, we believe that the use of the poly(lactic acid)-based carrier will provide easier handling of cell sheets and boost the development of the field of regenerative medicine.

## Disclosure of Potential Conflicts of Interest

No potential conflicts of interest were disclosed.
